# Comprehensive cancer-gene panels can be used to estimate mutational load and predict clinical benefit to PD-1 blockade in clinical practice

**DOI:** 10.18632/oncotarget.5950

**Published:** 2015-10-01

**Authors:** Luís Felipe Campesato, Romualdo Barroso-Sousa, Leandro Jimenez, Bruna R. Correa, Jorge Sabbaga, Paulo M. Hoff, Luiz F. L. Reis, Pedro Alexandre F. Galante, Anamaria A. Camargo

**Affiliations:** ^1^ Instituto Ludwig de Pesquisa sobre o Câncer, São Paulo, Brazil; ^2^ Hospital Sírio-Libanês, São Paulo, Brazil; ^3^ Departmento de Bioquímica, Instituto de Química, Universidade de São Paulo, São Paulo, Brazil

**Keywords:** cancer-gene panels, mutational load, PD-1 blockade, immunotherapy, response prediction

## Abstract

Cancer gene panels (CGPs) are already used in clinical practice to match tumor's genetic profile with available targeted therapies. We aimed to determine if CGPs could also be applied to estimate tumor mutational load and predict clinical benefit to PD-1 and CTLA-4 checkpoint blockade therapy. Whole-exome sequencing (WES) mutation data obtained from melanoma and non-small cell lung cancer (NSCLC) patients published by Snyder et al. 2014 and Rizvi et al. 2015, respectively, were used to select nonsynonymous somatic mutations occurring in genes included in the Foundation Medicine Panel (FM-CGP) and in our own Institutional Panel (HSL-CGP). CGP-mutational load was calculated for each patient using both panels and was associated with clinical outcomes as defined and reported in the original articles. Higher CGP-mutational load was observed in NSCLC patients presenting durable clinical benefit (DCB) to PD-1 blockade (FM-CGP *P*=0.03, HSL-CGP *P*=0.01). We also observed that 69% of patients with high CGP-mutational load experienced DCB to PD-1 blockade, as compared to 20% of patients with low CGP-mutational load (FM-CGP and HSL-CGP *P*=0.01). Noteworthy, predictive accuracy of CGP-mutational load for DCB was not statistically different from that estimated by WES sequencing (*P*=0.73). Moreover, a high CGP-mutational load was significantly associated with progression-free survival (PFS) in patients treated with PD-1 blockade (FM-CGP *P*=0.005, HR 0.27, 95% IC 0.105 to 0.669; HSL-CGP *P*=0.008, HR 0.29, 95% IC 0.116 to 0.719). Similar associations between CGP-mutational load and clinical benefit to CTLA-4 blockade were not observed. In summary, our data reveals that CGPs can be used to estimate mutational load and to predict clinical benefit to PD-1 blockade, with similar accuracy to that reported using WES.

## INTRODUCTION

Checkpoint blockade immunotherapy, which targets regulatory pathways in T-cells to enhance antitumor immune responses, is revolutionizing cancer treatment. Three monoclonal antibodies directed to two different immune checkpoint molecules - CTLA-4 (cytotoxic T-lymphocyte antigen 4) and PD-1 (programmed death receptor 1) − have now been approved by the U.S. Food and Drug Administration for treatment of melanoma and NSCLC patients [1,2]. Although significant and durable response rates have been reported, clinical benefit of these inhibitors has been limited to a subset of patients and has not been observed in all tumor types [3,4], highlighting the need for identification of predictive biomarkers for patient stratification and selection.

Human tumors typically harbor different types of somatic mutations, including nonsynonymous mutations that alter amino acid residues of a protein. Altered amino acid residues resulting from nonsynonymous mutations can create new T-cell epitopes (neoepitopes) from a previously self-peptide [5-7], serving as neoantigens capable of eliciting an antitumor immune response. Neoantigen formation is thus a probabilistic event, where each nonsynonymous mutation increases the chances of immunogenic neoantigen formation. Therefore, tumor mutational load, defined as the number of nonsynonymous mutations in the tumor, could be used as a predictive marker for checkpoint blockade immunotherapy. Indeed, emerging data suggest that recognition of such neoantigens is a major factor in the activity of immunotherapies. In addition, tumor mutational load estimated by WES has proven to be directly associated with clinical benefit to immune checkpoint blockade [8-10].

In a recent report, Snyder et al. [8] showed that tumor mutational load, estimated by WES, was higher in melanoma patients presenting long-term clinical benefit to CTLA-4 blockade when compared to patients with minimal or no clinical benefit. In addition, they observed that a high mutational load was significantly correlated with improved overall survival (OS) in a discovery cohort and with a non-significant trend towards improved OS in the validation cohort. Rizvi et al. [9] also reported a significant association between tumor mutational load, estimated by WES, and DCB in NSCLC patients treated with PD-1 blockade. A significant direct association between high mutational load and PFS was also reported. In addition, associations between high mutational load, DCB and PFS were reproduced in an independent validation cohort [9]. Finally, Le et al. [10] analyzed 41 patients with progressive metastatic carcinomas with mismatch repair deficiency (MRD) or mismatch repair proficiency (MRP) treated with PD-1 blockade. MRD tumors have 10 to 100 times more somatic mutations than MRP tumors and these tumors frequently contain prominent lymphocyte infiltrates, indicating an active immune response [11,12]. Objective response rates and PFS were significantly higher in patients with MRD colorectal tumors and MRD non-colorectal tumors. Together, these studies demonstrated that a high tumor mutational load, estimated by WES, may be used to predict response to immune checkpoint blockade therapy.

However, WES is not yet routinely available in the clinical practice, due to its high cost and time-consuming analysis. On the other hand, cancer-gene panels (CGPs) composed by ~300-600 well-characterized cancer-related genes are now commonly used in the clinics to match tumor's genetic profile with available targeted therapies. By analyzing matched tumor-normal DNA, CGPs can accurately filter rare germline variants and enable unambiguous identification of somatic mutations in therapeutically relevant cancer-genes in a single assay at a reasonable cost and timeframe [13,14]. Therefore, in the present study we sought to determine if CGPs could be used to accurately estimate mutational load and to predict response to immune checkpoint blockade, without the need of tumor WES.

## RESULTS

### CGP-mutational load is associated with clinical benefit to PD-1 blockade therapy in NSCLC patients

**Figure 1:**
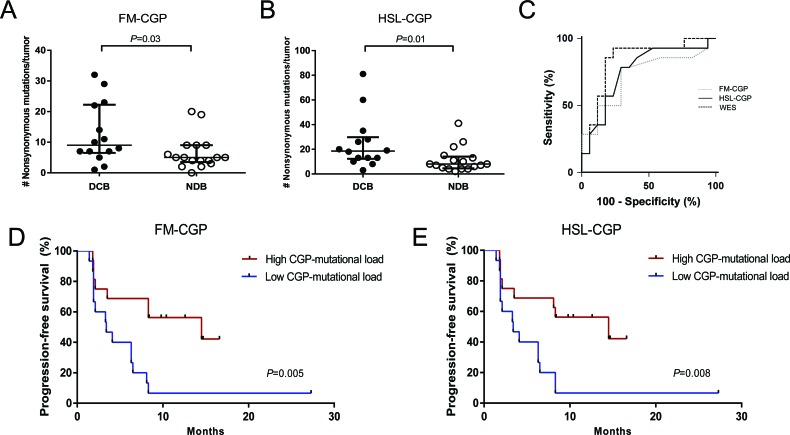
CGPs-mutational load is significantly associated with clinical benefit of anti-PD-1 therapy in NSCLCs **A.** FM-CGP mutational load in patients with DCB (*n* = 14) compared to those with NDB (n = 17) (median 9 versus 5, Mann-Whitney *P* = 0.03). **B.** HSL-CGP mutational load in patients with DCB (*n* = 14) compared to those with NDB (*n* = 17) (median 18.5 versus 8, Mann-Whitney *P* = 0.01). **C.** Receiver operation characteristic analysis (ROC) curves using FM-CGP, HSL-CGP and WES as predictors of DCB (*P* = 0.73). **D.** PFS in tumors with high CGP-mutational load (*n* = 16) compared to those with low CGP mutational load (*n* = 15) in FM-CGP (HR 0.26, 95% CI 0.10-0.67, Log-rank *P* = 0.005). **E.** PFS in tumors with high CGP-mutational load (*n* = 16) compared to those with low CGP mutational load (*n* = 15) in HSL-CGP (HR 0.29, 95% CI 0.11-0.72, Log-rank *P* = 0.008). In **A.** and **B.**, median and interquartile ranges of nonsynonymous mutations for each panel are shown, with individual values for each tumor shown with dots.

Whole-exome sequencing (WES) mutation data obtained from melanoma [8] and non-small cell lung cancer (NSCLC) patients [9] were used to select nonsynonymous somatic mutations occurring in genes included in the Foundation Medicine Panel (FM-CGP, Table S1) and in our own Institutional Panel (HSL-CGP, Table S2). Mutational load estimated using either one of the two panels was significantly associated with DCB in NSLC patients treated with PD-1 blockade. The median number of nonsynonymous somatic mutations calculated using FM-CGP was 9 and 5 for tumors from patients with DCB and NDB, respectively (Mann-Whitney *P* = 0.03, Figure 1A and Table S3). For the HSL-CGP, the median number of nonsynonymous somatic mutations was 18.5 and 8 for tumors from patients with DCB and NDB, respectively (Mann-Whitney *P* = 0.01, Figure 1B and Table S4).

Patients were then grouped, according to their number of nonsynonymous somatic mutations, in a high (≥7 for FM-CGP and ≥13 for HSL-CGP) and a low CGP-mutational load group (<7 for FM-CGP and <13 for HSL-CGP). DCB rates and PFS were significantly greater in patients with a high CGP-mutational load. We observed that 69% of patients with high CGP-mutational load, determined by either one of the two panels, presented DCB. In contrast, DCB was observed in only 20% of patients with low CGP-mutational load (Fisher's exact test *P* = 0.01 for FM-CGP and HSL-CGP, Table 1). Noteworthy, predictive accuracy of CGPs-mutational load for DCB was not statistically different to that estimated by WES sequencing (*P* = 0.73, Figure 1C). All three ROC curves presented similar AUC, sensitivity and specificity (Table 2). Finally, a high mutational load calculated by both CGPs was also significantly associated with PFS (median PFS 14.5 versus 3.4 months, Log-rank *P* = 0.005, HR 0.27, 95% IC 0.105 to 0.669 for the FM-CGP and median PFS 14.5 versus 3.4 months, Log-rank *P* = 0.008, HR 0.29, 95% IC 0.116 to 0.719 for the HSL-CGP, Figure 1D-E).

**Table 1:** CGP-mutational load is associated with clinical benefit to PD-1 blockade in NSCLC patients

Cancer-Gene Panel	CGP Mutational Load	Cutoff	n	Median mutations/sample (interquartile range)	Number of DCB patients n(%)	Fisher's Exact P	Median PFS (months)	Log-Rank P	HR for PFS (95% CI)
**FM-Panel**	High load	**7** mutations	16	10.5(8.7-20.5)	11(68.8)	0.01	14.5	0.005	0.265 (0.105 to 0.669)
	Low load		15	4(2-5)	3(20)		3.4		
**HSL-Panel**	High load	**13** mutations	16	21(14.5-29.7)	11(68.8)	0.01	14.5	0.008	0.288 (0.116 to 0.719)
	Low load		15	7(4-8)	3(20)		3.4		

**Table 2:** Comparison of ROC curves

Gene Panel	AUC	Median number of mutations	Sensitivity (%)	Specificity (%)	*P*-value
**FM-CGP**	0.73	7	78.6	70.6	0.02
**HSL-CGP**	0.76	13	78.6	70.6	0.004
**WES**	0.84	190	85.7	76.6	0.0001

Comparison between ROC Curves was carried out as previously reported by Datong et al., 1988 [21].

Pairwise comparison: FM-CGP vs. HSL-CGP, *P*= 0.79; FM-CGP vs. WES, *P*=0.44; HSL-CGP vs. WES, *P*=9.57

### CGP-mutational load is not associated with clinical benefit to CTLA-4 blockade therapy in melanoma patients

**Figure 2:**
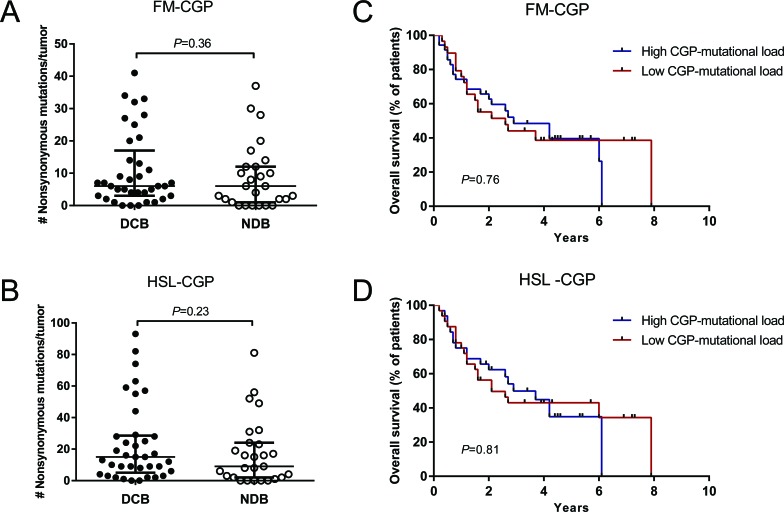
CGPs-mutational load is not associated with clinical benefit of anti-CTLA-4 therapy in Melanomas **A.** FM-CGP mutational load in patients with DCB (*n* = 37) compared to those with NDB (*n* = 27) (median 6, Mann-Whitney *P* = 0.36). **B.** HSL-CGP mutational load in patients with DCB (*n* = 37) compared to those with NDB (*n* = 27) (median 15 versus 8.5, Mann-Whitney *P* = 0.23). **C.** PFS in tumors with high CGP-mutational load (*n* = 30) compared to those with low nonsynonymous burden (*n* = 34) in FM-CGP (HR 1.10, 95% CI 0.57-2.11, Log-rank *P* = 0.76). **D.** PFS in tumors with higher nonsynonymous mutation burden (*n* = 29) compared to those with low CGP mutational load (*n* = 35) in HSL-CGP (HR 1.08, 95% CI 0.57-2.05, Log-rank *P* = 0.81). In **A.** and **B.**, median and interquartile ranges of nonsynonymous mutations for each panel are shown as horizontal lines, with individual values for each tumor shown as dots.

We did not observe a significant association between FM and HSL-CGP-mutational loads and DCB in melanoma patients treated with CTLA-4 blockade. The median number of nonsynonymous somatic mutations calculated using the FM-CGP was 6 for tumors from patients with DCB and with minimal or no clinical benefit (Mann-Whitney *P* = 0.37, Figure 2A and Table S5). Meanwhile, the HSL-CGP median number of nonsynonymous somatic mutations was 15 and 9 for tumors from patients with DCB and minimal or no clinical benefit, respectively (Mann-Whitney *P* = 0.24, Figure 2B and Table S6).

Patients were again grouped according to the number of mutations in a high and a low CGP-mutational load group. Subsequently, DCB rates and OS were determined for both groups. Durable clinical benefit rates were not associated with mutational load estimated using both FM-CGP (Fisher's exact test *P* = 0.80, Table S7) and HSL-CGP (Fisher's exact test *P* = 1.00, Table S7). Moreover, no differences in OS were observed for patients in the high and low CGP-mutational load groups, irrespectively of the panel used (Figure 2C-2D).

## DISCUSSION

The catalogue of cancer types therapeutically manageable with immune checkpoint blockade therapy is growing at an astonishing pace. However, less than half of all patients respond to these treatments [3,4]. Identification of predictive biomarkers for patient stratification is a pressing issue, since it would minimize unnecessary exposure of patients to potentially life-threatening immune-related toxicities, as well as reduce the financial costs imposed on health systems by expensive drugs.

Some studies have established partial correlations between outcomes with CTLA-4 blockade and peripheral-blood lymphocyte count, markers of T-cell activation, an “inflammatory” tumor microenvironment and maintenance of high-frequency T-cell receptor clonotypes precluding their utilization in clinics [15-17]. Other studies have reported that pre-treatment programmed cell death ligand-1 (PD-L1) expression in tumor biopsy directly correlate with response to anti-PD-1 therapies, but many tumors scored as PD-L1 positive do not respond, while some responses occur in PD-L1-negative tumors [18-20]. These controversial results probably reflect the sum of technical issues related to immunohistochemistry staining and scoring system, as well as to biologic issues related to PD-L1 expression on other cells present in the tumor microenvironment, including infiltrating myeloid cells.

Recent studies, however, demonstrated that tumors with a high load of nonsynonymous somatic mutations determined by WES are more likely to respond to checkpoint blockade immunotherapy, as in theory these tumors would have a higher diversity of neoantigens that could trigger an immune response when the CTLA-4/PD-1 inhibition is bypassed [8-10]. Nevertheless, WES and neoantigen identification is not yet a routine in the clinical practice and more straightforward and affordable approaches to estimate tumor mutational load and predict response to checkpoint blockade are needed.

Here, we demonstrated that the association between mutational load and clinical benefit to PD-1 blockade is also observed when CGPs are used to estimate mutation burden. Noteworthy, predictive accuracy is apparently lost when smaller CGPs (<150 cancer-genes) are used to estimate mutational loads, indicating that comprehensive gene panels, composed of >300 cancer-genes, should be employed (Figure S1, Table S8-10). Most importantly, we demonstrated that comprehensive CGPs can be used to predict response to PD-1 blockade in clinics with similar accuracy to results reported by Rizvi and colleagues [9].

Our analysis does not support a similar use for cancer-gene panels in the prediction of response to CTLA-4. This result, however, is not totally unexpected since blockade of CTLA-4 and PD-1 receptors present distinct mechanisms of action and the association between mutational load and clinical benefit also seems to be weaker for CTLA-4 blockade when compared to PD-1 blockade, as previously reported [8]. Although Snyder and colleagues have observed a significant association between mutational load and overall survival in the discovery cohort, a similar result was not reproduced in the validation cohort, indicating that mutational load alone is not sufficient to predict clinical benefit to CTLA-4 blockade.

Even though all these studies are limited by the small number of patients analyzed, our results are encouraging, considering the broader therapeutic activity of PD-1 blockade alone or its use in combination with other therapies. Since CGPs are already routinely used in many cancer centers to guide therapeutic choices, our findings will certainly accelerate the validation and the implementation of mutational load estimates as a clinically useful predictive marker, further extending the current benefits of immune checkpoint blockade.

## MATERIALS AND METHODS

### Study design

We downloaded WES mutation data from melanoma and NSCLC patients published by Snyder et al. [8] and Rizvi et al. [9], respectively, and selected nonsynonymous somatic mutations occurring in genes included in the Foundation Medicine Panel (FM-CGP; Table S1), as well as in a CGP developed at our own Institution (HSL-CGP; Table S2). In both original studies matched normal DNA, extracted from peripheral blood, was used to filter germline variants and to enable unambiguous identification of somatic mutations. Next, we calculated the CGP-mutational load for each patient enrolled in these two studies using just mutated genes present in FM and HSL CGPs. CGP-mutational loads were then associated with clinical outcomes as defined and reported in the original articles [8, 9]. Patients included in discovery and validation cohorts in the original articles were grouped in a single larger cohort to increase the statistical power of our analysis.

### Statistical analysis

NSCLC patients with partial or stable response lasting >6 months were classified as DCB, according to the original article [9]. For the purpose of this manuscript, melanoma patients presenting radiographic evidence of freedom from disease or evidence of a stable or decreased volume of disease for >6 months (described as long-term benefit patients in the original article [8]) were also classified as DCB. Mann-Whitney test was used to compare CGP-mutational loads between groups of patients with DCB and NDB (no durable benefit). Patients were then divided in a high CGP-mutational load group (defined as those equal or above the median mutational load of the cohort) and low CGP-mutational load group (defined as those below the median mutational load of the cohort). The proportion of patients with DCB and NDB in each group was compared using Fisher's exact test. Log-rank test was used to compare Kaplan-Meier survival curves for each group. All these statistical analyses were performed using GraphPad Prism v.6 (Graphpad Prism Software, San Diego, CA). Receiver operating characteristic (ROC) curve analysis was used to discriminate DCB and NDB patients. The area under the curve (AUC), sensitivity and specificity for each CGP and WES were determined using MedCalc software (version 15.6.1). Comparison between AUC values was carried out according to Delong et al. [21] using STATA software (version 13.0).

## SUPPLEMENTARY MATERIAL TABLES AND FIGURE


