# Supplementary material for: Comprehensive cancer-gene panels can be used to estimate mutational load and predict clinical benefit to PD-1 blockade in clinical practice
Source: Oncotarget. 2015 Oct 1;6(33):34221–7. doi: 10.18632/oncotarget.5950 (PMC4741447; doi:10.18632/oncotarget.5950)
Supplement: Supplementary file 1 [file oncotarget-06-34221-s001.pdf]

Comprehensive cancer-gene panels can be used to estimate mutational load and predict clinical benefit to PD-1 blockade in clinical practice

Supplementary Material

Table of contents

Table S1. FM-CGP Genes **pg. 2**

Table S2. HSL-CGP Genes **pg. 3**

Table S3. NSCLC Data – FM-CGP **pg. 5**

Table S4. NSCLC Data – HSL-CGP **pg. 6**

Table S5. Melanoma Data – FM-CGP **pg. 7**

Table S6. Melanoma Data – HSL-CGP **pg. 8**

Table S7. CGP-mutational load x clinical response to CTLA-4 blockade **pg. 9**

Table S8. NSCLC Data – Cancer SelectR 88-CGP **pg. 10**

Table S9. NSCLC Data – Cancer SelectR 203-CGP **pg. 11**

Table S10. NSCLC Data – Qiagen-CGP **pg. 12**

Figure S1. Clinical benefit prediction by smaller CGPs **pg. 13**

Table S1. FM-CGP genes.

GeneSymbol – FM-CGP								
ABL1	BRIP1	CTCF	FGF10	GSK3B	KRAS	NOTCH2	PTCH1	SOX2
ABL2	BTG1	CTNNA1	FGF14	H3F3A	LMO1	NOTCH3	PTEN	SOX9
ACVR1B	BTK	CTNNB1	FGF19	HGF	LRP1B	NPM1	PTPN11	SPEN
AKT1	EMSY	CUL3	FGF23	HNF1A	LYN	NRAS	QKI	SPOP
AKT2	CARD11	CYLD	FGF3	HRAS	LZTR1	NSD1	RAC1	SPTA1
AKT3	CBFB	DAXX	FGF4	HSD3B1	MAGI2	NTRK1	RAD50	SRC
ALK	CBL	DDR2	FGF6	HSP90AA1	MAP2K1	NTRK2	RAD51	STAG2
APC	CCND1	DICER1	FGFR1	IDH1	MAP2K2	NTRK3	RAF1	STAT3
AR	CCND2	DNMT3A	FGFR2	IDH2	MAP2K4	NUP93	RANBP2	STAT4
ARAF	CCND3	DOT1L	FGFR3	IGF1R	MAP3K1	PAK3	RARA	STK11
ARFRP1	CCNE1	EGFR	FGFR4	IGF2	MCL1	PALB2	RB1	SUFU
ARID1A	CD274	EP300	FH	IKBKE	MDM2	PARK2	RBM10	SYK
ARID1B	CD79A	EPHA3	FLCN	IKZF1	MDM4	PAX5	RET	TAF1
ARID2	CD79B	EPHA5	FLT1	IL7R	MED12	PBRM1	RICTOR	TBX3
ASXL1	CDC73	EPHA7	FLT3	INHBA	MEF2B	PDCD1LG2	RNF43	TERC
ATM	CDH1	EPHB1	FLT4	INPP4B	MEN1	PDGFRA	ROS1	TERT
ATR	CDK12	ERBB2	FOXL2	IRF2	MET	PDGFRB	RPTOR	TET2
ATRX	CDK4	ERBB3	FOXP1	IRF4	MITF	PDK1	RUNX1	TGFBR2
AURKA	CDK6	ERBB4	FRS2	IRS2	MLH1	PIK3C2B	RUNX1T1	TNFAIP3
AURKB	CDK8	ERG	FUBP1	JAK1	MPL	PIK3CA	SDHA	TNFRSF14
AXIN1	CDKN1A	ERRFI1	GABRA6	JAK2	MRE11A	PIK3CB	SDHB	TOP1
AXL	CDKN1B	ESR1	GATA1	JAK3	MSH2	PIK3CG	SDHC	TOP2A
BAP1	CDKN2A	EZH2	GATA2	JUN	MSH6	PIK3R1	SDHD	TP53
BARD1	CDKN2B	AMER1	GATA3	KAT6A	MTOR	PIK3R2	SETD2	TSC1
BCL2	CDKN2C	FAM46C	GATA4	KDM5A	MUTYH	PLCG2	SF3B1	TSC2
BCL2L1	CEBPA	FANCA	GATA6	KDM5C	MYC	PMS2	SLIT2	TSHR
BCL2L2	CHD2	FANCC	c17orf39	KDM6A	MYCL	POLD1	SMAD2	U2AF1
BCL6	CHD4	FANCD2	GLi1	KDR	MYCN	POLE	SMAD3	VEGFA
BCOR	CHEK1	FANCE	GNA11	KEAP1	MYD88	PPP2R1A	SMAD4	VHL
BCORL1	CHEK2	FANCF	GNA13	KEL	NF1	PRDM1	SMARCA4	WISP3
BLM	CIC	FANCG	GNAQ	KIT	NF2	PREX2	SMARCB1	WT1
BRAF	CREBBP	FANCL	GNAS	KLHL6	NFE2L2	PRKAR1A	SMO	XPO1
BRCA1	CRKL	FAS	GPR124	KMT2A	NFKBIA	PRKCI	SNCAIP	ZBTB2
BRCA2	CRLF2	FAT1	GRIN2A	KMT2C	NKX2-1	PRKDC	SOCS1	ZNF217
BRD4	CSF1R	FBXW7	GRM3	KMT2D	NOTCH1	PRSS8	SOX10	ZNF703

Table S2. HSL-CGP genes.

GeneSymbol – HSL-CGP								
AARS2	ARID1A	BRIP1	CDKN2A	CUL3	ERG	FH	GOT2	INHBA
ABCB1	ARID1B	BTG1	CDKN2B	CUL4B	ERRFI1	FIGF	GPHN	INPP4B
ABCC1	ARID2	BTK	CDKN2C	CUX1	ESR1	FKBP9	GPR124	INPPL1
ABL1	ARID5B	BUB1B	CEBPA	CYLD	EWSR1	FLCN	GPS2	INTS12
ABL2	ASPM	C11orf30	CEP76	DAXX	EZH1	FLG	GRIN2A	IPO7
ACO1	ASXL1	C3orf70	CFH	DDR2	EZH2	FLT1	GRM3	IRF2
ACVR1B	ASXL2	CAD	CHD2	DDX3X	EZR	FLT3	GSK3B	IRF4
ACVR2B	ATG13	CAP2	CHD4	DDX5	FAM123B	FLT4	GTF2H1	IRF6
ADAM10	ATM	CARD11	CHD8	DHX9	FAM166A	FN1	GUSB	IRS2
ADCY1	ATP5B	CARM1	CHEK1	DIAPH1	FAM46C	FOXA1	H3F3A	ITGA6
ADNP	ATR	CASP8	CHEK2	DICER1	FANCA	FOXA2	H3F3C	ITGB7
ADORA2A	ATRX	CAST	CIC	DIS3	FANCC	FOXL2	HDAC4	ITPKB
AHNAK2	AURKA	CBFB	CLIP1	DMD	FANCD2	FOXP1	HGF	JAK1
AJUBA	AURKB	CBL	CLTC	DNAH12	FANCE	FOXQ1	HIST1H1E	JAK2
AKAP9	AXIN1	CCAR1	CLTCL1	DNER	FANCF	FRG1B	HIST1H3B	JAK3
AKD1	AXIN2	CCDC120	CNBD1	DNMT1	FANCG	FRMD7	HIST1H4E	JUN
AKT1	AXL	CCDC6	CNKSR1	DNMT3A	FANCL	FRS2	HLA-A	KALRN
AKT2	AZGP1	CCND1	COL18A1	DOCK2	FAS	FUBP1	HLA-B	KAT6A
AKT3	B2M	CCND2	COL5A1	DOT1L	FAT1	GABRA6	HMGXB4	KDM5A
ALK	BAP1	CCND3	COL5A3	EEF1A1	FBN2	GATA1	HNF1A	KDM5C
ALKBH6	BARD1	CCNE1	CREB1	EGFR	FBXO11	GATA2	HRAS	KDM6A
ALPK2	BCL2	CD1D	CREBBP	EIF2S2	FBXW7	GATA3	HSD3B1	KDR
AMPH	BCL2L1	CD274	CRKL	EIF4A2	FES	GATA4	HSP90AA1	KEAP1
ANAPC1	BCL2L2	CD70	CRLF2	ELF3	FGF10	GATA6	HSP90B1	KEL
ANK3	BCL6	CD79A	CRTC1	EP300	FGF14	GID4	HSPA8	KIAA1109
ANKRD12	BCLAF1	CD79B	CRTC3	EPHA2	FGF19	GLi1	HUWE1	KIFC3
APAF1	BCOR	CDC27	CSDE1	EPHA3	FGF23	GNA11	IDH1	KIT
APC	BCORL1	CDC73	CSF1R	EPHA5	FGF3	GNA13	IDH2	KLF4
APOL2	BHMT2	CDH1	CTCF	EPHA7	FGF4	GNAI1	IGF1R	KLHL6
AR	BLM	CDK12	CTNNA1	EPHB1	FGF6	GNAQ	IGF2	KLHL8
ARAF	BRAF	CDK4	CTNNB1	ERBB2	FGFBP1	GNAS	IGF2R	KRAS
ARFRP1	BRCA1	CDK6	CTNND1	ERBB2IP	FGFR1	GNB1	IKBKE	LCP1
ARHGAP32	BRCA2	CDK8	CTSL1	ERBB3	FGFR2	GNPTAB	IKZF1	LCTL
ARHGAP35	BRD4	CDKN1A	CTTN	ERBB4	FGFR3	GOLGA5	IL7R	LEPR
ARHGEF6	BRE	CDKN1B	CUL1	ERCC2	FGFR4	GOT1	ING1	LIFR

GeneSymbol – HSL-CGP (cont.)								
LMO1	MITF	NEB	PAPD5	PPP2R1A	RNF43	SMARCB1	TCP11L2	U2AF1
LPP	MLH1	NEDD4L	PARK2	PPP6C	ROS1	SMC1A	TDRD10	UHRF1BP1L
LRP1B	MLL	NF1	PARP1	PRDM1	RPGR	SMC3	TERC	VEGFA
LRRK2	MLL2	NF2	PARP4	PREX2	RPL22	SMO	TERT	VHL
LUM	MLL3	NFE2L2	PAX5	PRKAR1A	RPL5	SNCAIP	TET2	WASF3
LYN	MLL4	NFKBIA	PBRM1	PRKCD	RPS15	SNX25	TFDP1	WHSC1
LZTR1	MLLT10	NIN	PCBP1	PRKCE	RPS2	SOCS1	TGFBR2	WHSC1L1
MAGI2	MLLT3	NKX2-1	PCM1	PRKCI	RPSAP58	SOS1	TGIF1	WISP3
MALAT1	MLLT4	NLRP3	PDAP1	PRKDC	RPTOR	SOX10	TIMM17A	WRN
MAP2K1	MMP2	NONO	PDCD1LG2	PRPF8	RSBN1L	SOX17	TJP2	WT1
MAP2K2	MN1	NOS1	PDCD2L	PRSS8	RUNX1	SOX2	TNF	XIRP2
MAP2K4	MORC4	NOTCH1	PDGFRA	PSEN1	RUNX1T1	SOX9	TNFAIP3	XPO1
MAP3K1	MPL	NOTCH2	PDGFRB	PTCH1	RXRA	SPATA6	TNFRSF10C	ZBTB2
MAP3K3	MPO	NOTCH3	PDK1	PTEN	SACS	SPEN	TNFRSF14	ZFHX3
MAP3K4	MRE11A	NPM1	PDSS2	PTGS2	SDHA	SPOP	TNKS	ZFP36L1
MAP4K3	MSH2	NR1H2	PHF6	PTPN11	SDHB	SPTA1	TOM1	ZFP36L2
MAPK8IP1	MSH6	NR4A2	PHLDA1	PTPRC	SDHC	SRC	TOP1	ZMYM2
MAST2	MSN	NRAS	PIK3C2A	QKI	SDHD	SRSF2	TOP2A	ZNF132
MATK	MST1	NSD1	PIK3C2B	RAB40A	SELP	STAG2	TOP3A	ZNF180
MAX	MTOR	NTN3	PIK3CA	RAC1	SEPT12	STAT3	TP53	ZNF217
MBD1	MUC17	NTN4	PIK3CB	RAD21	SERPINB13	STAT4	TP53BP1	ZNF300
MCL1	MUTYH	NTRK1	PIK3CG	RAD50	SETD2	STK11	TPR	ZNF471
MDC1	MXRA5	NTRK2	PIK3R1	RAD51	SETDB1	STK19	TPX2	ZNF483
MDM2	MYB	NTRK3	PIK3R2	RAF1	SF3B1	STX2	TRAF3	ZNF620
MDM4	MYC	NUMA1	PIK3R5	RANBP2	SFRS2	SUFU	TRAF7	ZNF703
MECOM	MYCL	NUP210L	PIP5K1A	RANBP3	SGK1	SUZ12	TRIM23	ZNF750
MED12	MYCN	NUP214	PLCG1	RARA	SIN3A	SYK	TRIM7	ZNF814
MED13	MYD88	NUP93	PLCG2	RASA1	SIRT4	SYNE1	TRIO	ZNF844
MED17	MYH9	NUP98	PLEC	RB1	SLC1A3	TAF1	TRIP11	ZNF91
MED23	MYOCD	ODAM	PMS2	RBM10	SLC26A3	TAP1	TRRAP	ZRANB3
MEF2A	NAV3	OMA1	POLD1	RBMX	SLC44A3	TBC1D12	TSC1	
MEF2B	NBPF1	OR4A16	POLE	RET	SLC4A5	TBL1XR1	TSC2	
MEN1	NBPF10	OR52N1	POLR3A	RHEB	SLIT2	TBX3	TSHR	
MET	NCOA3	OTUD7A	POTEF	RHOA	SMAD2	TCEB1	TTLL9	
METTL14	NCOA4	PABPC1	POU2AF1	RICTOR	SMAD3	TCF12	TUBA1A	
MGA	NCOR1	PAK3	POU2F2	RIT1	SMAD4	TCF7	TXNDC8	
MICALCL	NCOR2	PALB2	PPM1D	RNF213	SMARCA4	TCF7L2	TXNIP	

Table S3. NSCLC Data (Rizvi et al. 2014) – FM-CGP.

Durable Clinical Benefit (DCB)		No Durable Benefit (NDB)	
Patient_ID	Mutation_Count	Patient_ID	Mutation_Count
SA9755	32	TU0428	20
HE3202	29	ZA6505	19
Y2087	14	MA7027	9
RI1933	23	JB112852	6
M4945	22	SR070761	5
CA9903	11	GR4788	5
KA3947	5	BL3403	5
SC0899	7	DM123062	4
AL4602	7	WA7899	9
DI6359	8	R7495	2
RH090935	10	LO3793	9
SB010944	2	RO3338	5
SC6470	7	LO5004	4
GR0134	1	NI9507	4
		AU5884	2
		VA1330	3
		VA7859	0

Table S4. NSCLC Data (Rizvi et al. 2014) – HSL-CGP.

Durable Clinical Benefit (DCB)		No Durable Benefit (NDB)	
Patient_ID	Mutation_Count	Patient_ID	Mutation_Count
SA9755	81	TU0428	41
HE3202	60	ZA6505	26
Y2087	20	MA7027	22
RI1933	28	JB112852	10
M4945	35	SR070761	8
CA9903	19	GR4788	11
KA3947	13	BL3403	6
SC0899	26	DM123062	5
AL4602	13	WA7899	15
DI6359	18	R7495	7
RH090935	13	LO3793	13
SB010944	8	RO3338	7
SC6470	10	LO5004	8
GR0134	3	NI9507	4
		AU5884	4
		VA1330	4
		VA7859	2

Table S5. Melanoma Data (Snyder et al. 2014) – FM-CGP.

Durable Clinical Benefit (DCB)		No Durable Benefit (NDB)	
Patient_ID	Mutation_Count	Patient_ID	Mutation_Count
CR1509	8	SD2051	3
CR9306	32	SD5038	10
CR0095	9	SD5934	4
CR4880	20	SD5118	1
CR7623	5	SD6494	10
CR3665	5	SD7357	30
CR9699	25	NR3156	0
SD0346	13	NR5784	0
SD6336	5	NR8727	0
SD1494	27	NR4949	9
SD2056	3	NR1867	2
CR04885	41	NR3549	12
CR06670	4	NR9341	0
CR22640	3	NR4810	8
CR6126	9	NR2137	17
CR6161	21	NR4018	2
CRNR0244	6	NR4045	14
CRNR2472	0	NR4083	6
CRNR4941	1	NR4631	20
LSD0167	4	NR6689	0
LSD2057	1	NR6721	3
LSD3484	14	NR6842	0
LSD4691	0	NR8815	37
LSD4744	33	NR9445	6
LSD6819	6	NR9449	12
LSDNR1120	7	NR9521	28
LSDNR1650	2	NR9765	2
LSDNR3086	2		
LSDNR9298	7		
NR9705	4		
PR03803	1		
PR11217	0		
PR4035	11		
PR4046	6		
PR4077	34		
PR4091	7		
PR4092	28		

Table S6. Melanoma Data (Snyder et al. 2014) – HSL-CGP.

Durable Clinical Benefit (DCB)		No Durable Benefit (NDB)	
Patient_ID	Mutation_Count	Patient_ID	Mutation_Count
CR1509	17	SD2051	4
CR9306	74	SD5038	15
CR0095	13	SD5934	8
CR4880	29	SD5118	2
CR7623	9	SD6494	32
CR3665	8	SD7357	52
CR9699	57	NR3156	0
SD0346	25	NR5784	0
SD6336	9	NR8727	0
SD1494	59	NR4949	23
SD2056	6	NR1867	2
CR04885	93	NR3549	24
CR06670	19	NR9341	1
CR22640	9	NR4810	16
CR6126	24	NR2137	31
CR6161	44	NR4018	9
CRNR0244	15	NR4045	19
CRNR2472	3	NR4083	8
CRNR4941	3	NR4631	49
LSD0167	10	NR6689	0
LSD2057	2	NR6721	6
LSD3484	28	NR6842	0
LSD4691	0	NR8815	81
LSD4744	63	NR9445	16
LSD6819	16	NR9449	17
LSDNR1120	15	NR9521	56
LSDNR1650	2	NR9765	3
LSDNR3086	2		
LSDNR9298	22		
NR9705	31		
PR03803	1		
PR11217	0		
PR4035	28		
PR4046	9		
PR4077	82		
PR4091	12		
PR4092	55		

Table S7. CGP-mutational load is not associated with clinical benefit to CTLA-4 blockade in melanoma patients.

Cancer-Gene Panel	CGP Mutational Load	Cutoff	n	Median mutations/sample (interquartile range)	Number of DCB patients n (%)	Fisher's Exact *P*	Median OS (Years)	Log-Rank *P*	HR for PFS (95% CI)
**FM Panel**	High load	**6** mutations	35	12 (8-27)	21 (60)	0.8	2.9	0.76	1.105 (0.578 to 2.111)
	Low load		29	2 (0-3.5)	16 (55.2)		2.6		
**HSL Panel**	High load	**14** mutations	32	28 (17.5-56)	19 (59.4)	1.00	2.9	0.81	1.080 (0.567 to 2.055)
	Low load		32	3 (1-9)	18 (56.2)		2.1		

Table S8. NSCLC Data (Rizvi et al. 2014) – Cancer SelectR 88-CGP.

Durable Clinical Benefit (DCB)		No Durable Benefit (NDB)	
Patient_ID	Mutation_Count	Patient_ID	Mutation_Count
SA9755	7	TU0428	9
HE3202	11	ZA6505	6
Y2087	4	MA7027	0
RI1933	4	JB112852	2
M4945	5	SR070761	1
CA9903	7	GR4788	3
KA3947	2	BL3403	1
SC0899	5	DM123062	2
AL4602	4	WA7899	0
DI6359	2	R7495	2
RH090935	3	LO3793	5
SB010944	1	RO3338	3
SC6470	2	LO5004	2
GR0134	1	NI9507	3
		AU5884	2
		VA1330	2
		VA7859	0

Table S9. NSCLC Data (Rizvi et al. 2014) – Cancer SelectR 203-CGP.

Durable Clinical Benefit (DCB)		No Durable Benefit (NDB)	
Patient_ID	Mutation_Count	Patient_ID	Mutation_Count
SA9755	20	TU0428	14
HE3202	18	ZA6505	12
Y2087	7	MA7027	2
RI1933	13	JB112852	3
M4945	13	SR070761	5
CA9903	7	GR4788	4
KA3947	2	BL3403	4
SC0899	9	DM123062	4
AL4602	5	WA7899	2
DI6359	2	R7495	2
RH090935	5	LO3793	7
SB010944	1	RO3338	5
SC6470	3	LO5004	2
GR0134	1	NI9507	3
		AU5884	2
		VA1330	2
		VA7859	0

Table S10. NSCLC Data (Rizvi et al. 2014) – Qiagen-CGP.

Durable Clinical Benefit (DCB)		No Durable Benefit (NDB)	
Patient_ID	Mutation_Count	Patient_ID	Mutation_Count
SA9755	17	TU0428	14
HE3202	17	ZA6505	12
Y2087	7	MA7027	4
RI1933	7	JB112852	3
M4945	12	SR070761	5
CA9903	7	GR4788	3
KA3947	4	BL3403	3
SC0899	6	DM123062	2
AL4602	6	WA7899	3
DI6359	2	R7495	2
RH090935	6	LO3793	7
SB010944	1	RO3338	5
SC6470	4	LO5004	2
GR0134	0	NI9507	3
		AU5884	2
		VA1330	1
		VA7859	0

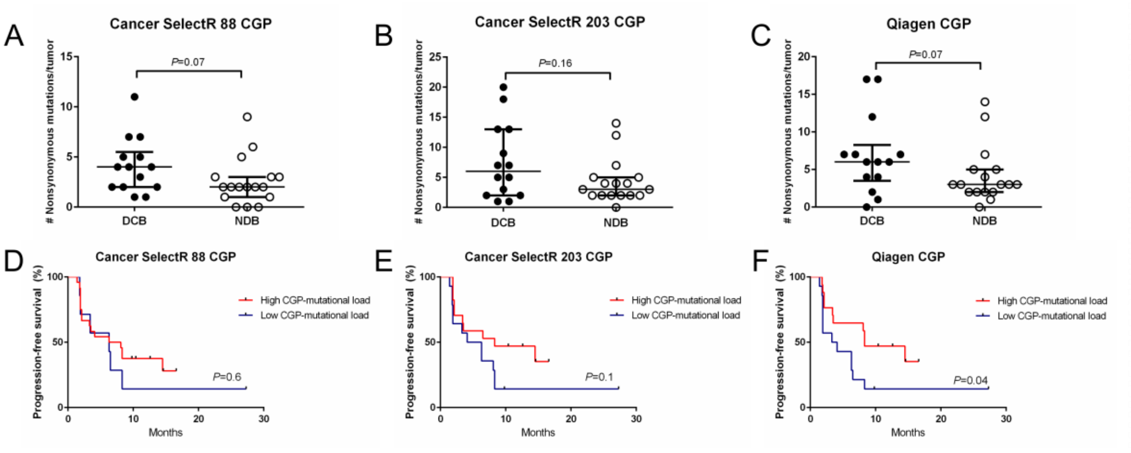

Figure S1. Smaller CGPs are not as accurate to determine clinical benefit of anti-PD-1 therapy in NSCLCs. (A) Cancer SelectR 88-CGP (76 cancer-genes, from PGDx) mutational load in patients with DCB (n=14) compared to those with NDB (n=17) (median 4 versus 2, Mann-Whitney *P*=0.07). **(B)** Cancer SelectR 203-CGP (195 cancer-genes, from PGDx) mutational load in in patients with DCB (n=14) compared to those with NDB (n=17) (median 6 versus 3, Mann-Whitney *P*=0.16). **(C)** Human Comprehensive Cancer GeneRead Targeted CGP (with 160 cancer-genes, from Qiagen) mutational load in patients with DCB (n=14) compared to those with NDB (n=17) (median 6 versus 3, Mann-Whitney *P*=0.07). **(D)** PFS in tumors with high CGP-mutational load compared to those with low CGP mutational load (Cutoff≥2) in Cancer SelectR 88-CGP (HR 0.71, 95% CI 0.25-2.04, Log-rank *P*=0.5). **(E)** PFS in tumors with high CGP-mutational load compared to those with low CGP mutational load (Cutoff≥4) in Cancer SelectR 203-CGP (HR 0.47, 95% CI 0.19-1.15, Log-rank *P*=0.1). **(F)** PFS in tumors with high CGP-mutational load compared to those with low CGP mutational load (Cutoff≥4) in Qiagen-CGP (HR 0.39, 95% CI 0.15-0.98, Log-rank *P*=0.04).
